# Atlas Hypoplasia and Ossification of the Transverse Atlantal Ligament: A Rare Cause of Cervical Myelopathy

**DOI:** 10.1155/2012/893284

**Published:** 2012-12-05

**Authors:** Rakan Bokhari, Saleh Baeesa

**Affiliations:** Division of Neurological Surgery, College of Medicine, King Abdulaziz University, P.O. Box 80215, Jeddah 21589, Saudi Arabia

## Abstract

Myelopathy at the level of the atlas is rarely encountered by the practicing spine surgeon. Due to the region's unique anatomy, compression of the cord at this level is either caused by a large compressing lesion or an abnormally stenotic canal. We describe a rare instance of a congenitally stenotic canal due to a hypoplastic intact posterior arch of atlas, coexisting with an extremely rare ossified transverse ligament of the atlas. The coexistence of these two lesions has only been documented thrice before. We describe the clinical presentation, imaging findings, and favorable response to surgery.

## 1. Introduction

Hypoplastic intact posterior arches of the atlas and symptomatic calcification of the transverse atlantoid ligament are very rarely seen at the craniovertebral junction, Having only been reported 17 and 15 times, respectively, in the literature [1–3].

We present a case of cervical myelopathy attributable to atlas posterior arch hypoplasia and an ossified transverse ligament, whose coexistence led to the slow development of cord compression and myelopathy. We present the radiologic appearance of these lesions and surgical management, to which the patient responded favorably. We also conducted a literature review and an attempt to explain why these may have coincided in our case.

This is the fourth such case in the literature, with two being identical to ours having both lesions concurrent [3, 4], while the more recent case had a further additional hypertrophied odontoid process contributing to the myelopathy [1].

## 2. Case Report

**Figure 1:**
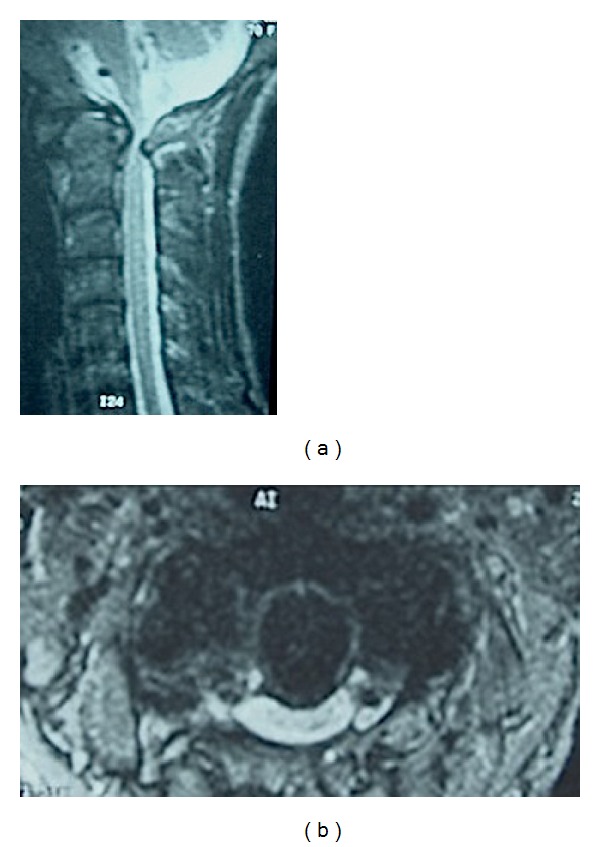
Sagittal (a) and axial (b) T2-weighted image MRI scans of the cervical spine demonstrating hypoplastic posterior arch of the atlas causing severe canal stenosis and spinal cord compression.

**Figure 2:**
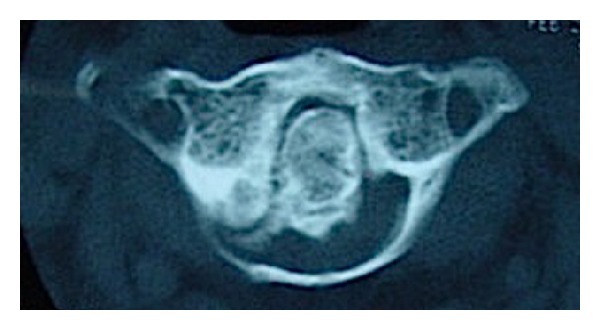
Axial CT scan at the level of the atlas demonstrating severe narrowing of the spinal canal and ossified transverse atlantal ligament.

A 68-year-old Saudi female presented with progressive deterioration of her walking and gait balance for 5 months. She has long history of cervicalgia for several years. There was no history of head or neck trauma or rheumatological diseases. General physical examination was normal, without cervical spine tenderness or limitation of range of motion. Neurological examination revealed normal cognitive function and cranial nerves. She had significant quadriparesis, where she was not able to raise her arm above her shoulders level and was unable to stand. Her weakness was more marked on the right side that was associated with hypoesthesia of the upper extremities, hyperreflexia in all limbs, and positive Babinski's sign. Routine laboratory tests, including CBC, electrolytes, renal, and hepatic profiles, were within normal. Her cervical X-rays revealed multiple degenerative disk diseases without evidence of instability on dynamic views. Magnetic resonance imaging (MRI) of the cervical spine revealed severe canal stenosis at the level of the atlas with significant cord compression (Figures 1(a) and 1(b)). The posterior arch of the atlas was absent and there was thickening of the transverse ligament. Computed tomography (CT) scan demonstrated severe canal stenosis, hypoplastic posterior arch of atlas vertebra, and ossification of the transverse atlantal ligament (Figure 2). The patient underwent cervical laminectomy of the hypoplastic posterior arch of the atlas. There was marked ligamentous thickening, which was forming a fibrous band, which was resected. The patient had uneventful postoperative course. She was able to walk with mild assistance after 6 months of extensive physiotherapy.

## 3. Discussion

Cervical canal stenosis is rarely encountered at the level of the atlas. This is due to the region's anatomy protective against compression, with the canal diameter at the retrodental level being between 17 to 25 mm while the spinal cord diameter at that level is 10–12 mm in diameter [5]. Myelopathy at this level would therefore require that the compressing lesion attain a considerable size or that the bony canal dimensions be compromised (altered by virtue of trauma, intervention, or developmental anomaly).

Although the majority of anomalies at the level of atlas reported in the literature are defects of either arches of atlas [4], these rarely lead to cord compression, since they usually either increase the canal space (essentially acting as an autolaminectomy) or leave it unaltered with a few exceptions [6]. In contrast to these anomalies, hypoplasia of an intact posterior arch of the atlas does in fact decrease canal dimensions [7]. It should be stressed that hypoplasia of an intact arch of the atlas is an entity distinct from arch clefts with a different pathogenesis that may involve premature fusion of synchondroses, or facetal hypertrophy from abnormal rotator mechanics [4].

Hypoplasia of an intact posterior arch of the atlas as a cause of symptoms has only been reported 16 times in the literature [1]. Its presence has been noted to coincide with several syndromes, suggesting a genetic component to this anomaly. The syndromes reported include Down syndrome, Turner syndrome, achondroplasia, and gonadal dysgenesis among others [8]. Anomalies of the atlas also show evidence of familial clustering and ethnic predisposition [4, 9]. Although believed to be a disease exclusive to the Far East, two recently reported cases were Caucasian [6, 10], showing that although predominantly affecting Asian males, it is not exclusive to a specific race or sex.

 Presentation is usually in middle age, with the exception of two cases of juvenile onset, both of who were Caucasian [11]. The reason why a congenital anomaly would reveal itself so late is that the canal stenosis is not usually critical until another stressor further narrows the canal or stresses the spine. Subaxial spondylosis also limits its motion, causing hypermobility at the axial level to compensate and make it vulnerable to compression by accentuating the effects of stenosis at extremes of motion [11].

This stressor may be in the form of trauma, or the gradual degenerative changes of age-related spondyloarthropathy [11]. Although in other very rare instances, as in our case, ossification of the transverse atlantoid ligament was implicated [1, 3, 4]. 

The usual presentation is with myelopathy [11], and diagnosis is usually straightforward provided the clinician is aware of this disease. CT is useful to assess the spinal canal dimensions and to seek out coexisting spine anomalies or pathologies either in the atlas or below; these may be coincidental or may be participating in causing the canal stenosis. MRI is useful to look for neural element compression, signal changes, and assess for degenerative changes in the cervical spine, all of which may alter treatment or surgical strategy. 

Response in reported cases has been favorable to simple posterior decompression with a C-1 laminectomy. This is to address the canal stenosis, but since the anomaly is rarely the sole cause of the canal stenosis, the other stressors have to be addressed and if needed, surgically corrected [11]. 

Calcification and ossification of the transverse atlantoid ligament is a disease suggested to be part of the spectrum of vertebral ligament ossification [12]. A recent study observed a 25% prevalence of TAL calcification in patients with subaxial ossification of the posterior longitudinal ligament, a phenomenon fairly common in the Far East.

Causes of paravertebral ligament calcification are numerous and not different from those of ligament calcification elsewhere, these include insulin resistance, calcium pyrophosphate dihydrate deposition diseases, rheumatoid arthritis, advancing age, obesity, and perhaps a genetic predisposition [12]. Another cause specific to the TAL is chronic atlantoaxial instability [13]. 

Diagnosis of TAL calcification is by CT, showing a calcified retro-odontoid mass following the contour of the TAL. MRI would be useful for CVJP as it better displays the noncalcified portions in addition to neural element compression. Contrast is usually unnecessary; it may reveal an inflammatory element, especially when associated with arthritides [14].

The management of transverse ligament calcification should then be both targeted at the underlying cause of calcification (e.g., metabolic or AAI), the calcified ligament itself if symptomatic, and its effect (neural element compression) if present [13].

The natural history of this disease is similar to that of cervical spondylosis; it has been displayed in several instances that, with the continuous degenerative forces causing ligament injury, hypertrophy and ossification, these masses can attain considerable size thereby causing myelopathy [15]. We believe it was because of the hypoplastic atlas that the patient had presented before the disease would sufficiently progress.

We cannot come up with a reason as to why this is the first and only encounter our institution would have with these entities and why this is the first case to be reported in the whole of the Middle East and North Africa region. This could either be due to a unique characteristic of our demographic or simply due to unfamiliarity to this disease from the part of clinician, radiologist, or pathologist [13]. It has been stated that a pathologist unfamiliar with the preparation required to display ossification of the TAL or not requested to do so, may cause the crystals to dissolve and the diagnosis to be missed [13]. We have no numbers to quote regarding the prevalence of this disease in our country or region, nor are there, to our knowledge, any reports of this disease described in our literature, despite the ubiquity of its proposed risk factors (obesity, insulin resistance, and Asian descent) in our population [16].

A newly emerging concept is to regard TAL ossification, AAOA, and AAI all as, perhaps, part of the craniovertebral junction's peculiar spondylotic process [14, 16]. A radiographic study conducted showed that TAL calcification is associated with osteoarthritis of the anterior atlanto-odontoid (AO) joint, with both being possibly caused by the constant stress of atlantoaxial instability (AAI) [17]. This should cause us to regard TAL as a marker for possible occult AAI and work up the patient accordingly. It should be mentioned that with our case we are observing a developing association between TAL and posterior arch of atlas hypoplasia (PAAH) [1, 3, 4], the fact that four cases of these individually very rare lesions would coexist indicates a high probability of a common link. This was never proposed in the literature previously and may, indeed be a coincidence, but judging by their individual rarity, this is unlikely. The exact explanation is beyond us, but we propose that perhaps PAAH reflects a propensity of tissues to calcify (in this case the premature ossification of the developing vertebra's cartilage), another possible mechanism is that biomechanics are affected by the anteriorly migrated posterior arch, increasing stresses on the atlantoaxial joints and stressing the TAL. Further studies on the biomechanics of craniovertebral junctions in the absence of an altered posterior arch may aid in understanding this association. 

We regret the fact that we only realized the full scope of this anomaly in retrospect, with the patient lost to follow up. With the many associations in the reviewed literature, we would have been more vigilant in searching for them in the patient herself and her family. 

## 4. Conclusions

We present a rare cause of myelopathy in our population, with none of the reported cases being in natives of the MENA region, despite the ubiquity of its risk factors. Studies are showing that ossification of the transverse ligament of atlas, if incidentally found, is not to be considered of no significance, but as a marker for local degenerative changes or systemic syndromes. Its presence should entail a thorough search for the growing list of reported associations. A thorough understanding of its significance, pathophysiology, and natural history will only be possible with more cases being reported and studied; this is only possible by increasing awareness among the medical community.
